# An Elvitegravir Nanoformulation Crosses the Blood–Brain Barrier and Suppresses HIV-1 Replication in Microglia

**DOI:** 10.3390/v12050564

**Published:** 2020-05-20

**Authors:** Yuqing Gong, Kaining Zhi, Prashanth K. B. Nagesh, Namita Sinha, Pallabita Chowdhury, Hao Chen, Santhi Gorantla, Murali M. Yallapu, Santosh Kumar

**Affiliations:** 1 Department of Pharmaceutical Sciences, University of Tennessee Health Science Center, Memphis, TN 38163, USA; ygong4@uthsc.edu (Y.G.); nsinha2@uthsc.edu (N.S.); pchowdhu@uthsc.edu (P.C.); 2 Plough Center for Sterile Drug Delivery Solutions, University of Tennessee Health Science Center, Memphis, TN 38163, USA; kzhi@uthsc.edu; 3 Laboratory of Signal Transduction, Memorial Sloan Kettering Cancer Center, New York, NY 10065, USA; bhusettp@mskcc.org; 4 Department of Pharmacology, University of Tennessee Health Science Center, Memphis, TN 38163, USA; hchen3@uthsc.edu; 5 Department of Pharmacology & Experimental Neuroscience, University Nebraska Medical Center, Omaha, NE 68198, USA; sgorantla@unmc.edu; 6 Department of Immunology and Microbiology, University of Texas Rio Grande Valley, McAllen, TX 78504, USA

**Keywords:** HIV, antiretroviral therapy, elvitegravir, nanomedicine, microglia

## Abstract

Even with an efficient combination of antiretroviral therapy (ART), which significantly decreases viral load in human immunodeficiency virus type 1 (HIV-1)-positive individuals, the occurrence of HIV-1-associated neurocognitive disorders (HAND) still exists. Microglia have been shown to have a significant role in HIV-1 replication in the brain and in subsequent HAND pathogenesis. However, due to the limited ability of ART drugs to cross the blood–brain barrier (BBB) after systemic administration, in addition to efflux transporter expression on microglia, the efficacy of ART drugs for viral suppression in microglia is suboptimal. Previously, we developed novel poly (lactic-*co*-glycolic acid) (PLGA)-based elvitegravir nanoparticles (PLGA-EVG NPs), which showed improved BBB penetration in vitro and improved viral suppression in HIV-1-infected primary macrophages, after crossing an in vitro BBB model. Our objective in the current study was to evaluate the efficacy of our PLGA-EVG NPs in an important central nervous system (CNS) HIV-1 reservoir, i.e., microglia. In this study, we evaluated the cyto-compatibility of the PLGA-EVG NPs in microglia, using an XTT (2,3-bis-(2-methoxy-4-nitro-5-sulfophenyl)-2H-tetrazolium-5-carboxanilide) assay and cellular morphology observation. We also studied the endocytosis pathway and the subcellular localization of PLGA NPs in microglia, using various endocytosis inhibitors and subcellular localization markers. We determined the ability of PLGA-EVG NPs to suppress HIV-1 replication in microglia, after crossing an in vitro BBB model. We also studied the drug levels in mouse plasma and brain tissue, using immunodeficient *NOD scid gamma* (*NSG*) mice, and performed a pilot study, to evaluate the efficacy of PLGA-EVG NPs on viral suppression in the CNS, using an HIV-1 encephalitic (HIVE) mouse model. From our results, the PLGA-EVG NPs showed ~100% biocompatibility with microglia, as compared to control cells. The internalization of PLGA NPs in microglia occurred through caveolae-/clathrin-mediated endocytosis. PLGA NPs can also escape from endo-lysosomal compartments and deliver the therapeutics to cells efficiently. More importantly, the PLGA-EVG NPs were able to show ~25% more viral suppression in HIV-1-infected human-monocyte-derived microglia-like cells after crossing the in vitro BBB compared to the EVG native drug, without altering BBB integrity. PLGA-EVG NPs also showed a ~two-fold higher level in mouse brain and a trend of decreasing CNS HIV-1 viral load in HIV-1-infected mice. Overall, these results help us to create a safe and efficient drug delivery method to target HIV-1 reservoirs in the CNS, for potential clinical use.

## 1. Introduction

Antiretroviral therapy (ART) is the most effective treatment plan to prevent and control the progress of HIV-1 [1]. However, while ART treatment can decrease the blood viral load in HIV-1-positive individuals, HAND still occurs, even with the best combination of ART [2]. Up to 50% of people living with HIV/AIDS (PLWHA) who were treated with ART showed symptoms of HAND, while their blood viral load(s) was undetectable [3]. The appearance and persistence of HAND are mainly due to the entry of HIV-1 into the brain via the “Trojan horse” mechanism through infected monocytes/macrophages [4,5]. Monocytes/macrophages can be infected by HIV-1 and can function as viral reservoirs because they are capable of surviving with HIV-1 infection up to 21 days [6]. These HIV-1-infected monocytes/macrophages infiltrate into the brain and spread the virus to resident macrophages and microglia in the central nervous system (CNS) [7]. Furthermore, HIV-1 actively replicates in macrophages and microglia, providing persistent viral replication in the brain [8,9]. Although neurons are not likely to be infected by the HIV-1 virus due to an absence of CD4 receptors, active replication of HIV-1 in infected macrophages and microglia produces toxic components, including viral proteins and inflammatory cytokines and chemokines, which damage to neurons and ultimately cause HAND [10,11]. Individuals with HAND commonly show characteristics of dysfunctional and impaired judgment, memory, multitasking, and attention [12]. Additionally, HIV-1 infection in the brain affects not only cognitive functioning but also the opportunity for HIV-1 eradication [13]. Currently, the major issue in HAND treatment is the relatively low blood–brain barrier (BBB) penetration of ART drugs with a suboptimal drug concentration in the brain [14]. Most ART drugs show limited CNS penetration relative to plasma drug concentration, even with ritonavir (RTV) boosting [15,16].

Microglia have been shown to have a significant role in HAND and HIV-1 replication in the brain. Unlike macrophages, microglia stay within the brain parenchyma and not in the circulation [17]. Reports suggest high expressions of CD4, CXCR4, CCR3, and CCR5 on microglia, which make them vulnerable to HIV-1 infection [18,19,20]. Microglia can also be activated upon exposure to HIV-1, HIV-1-related proteins, or molecules excreted by HIV-1-infected cells [21]. After microglial activation, they release cytokines, chemokines, excitotoxins, prostaglandins, metabolites, and HIV-1 proteins [19]. The literature reported increased expression of pro-apoptotic cytokines and TNF-α in infected microglia cells [21]. The released TNF-α further opens a paracellular route for HIV-1 invasion across the BBB, resulting in facilitated entry of HIV-1 proteins and cytokines from the periphery. These factors disrupt neuroimmune homeostasis and ion homeostasis, e.g., calcium homeostasis, and contribute to the progression of neural injury and HAND [11,22]. More importantly, these viral proteins and cellular products excreted by microglia induce astrocyte dysfunction and neuronal apoptosis, which might be the main reason for HAND [23]. Therefore, the persistent HIV-1 infection in microglia is one of the root causes of HAND.

Because of microglia’s role in HIV-1 pathologies inside the brain, maintaining ART drug concentrations in microglia becomes very critical in controlling HAND. Unfortunately, microglia have been reported to have a high expression of many efflux transporters, including MRPs, P-gp [24], BCRP, ABCG2 [25], and SPGP [26]. However, many ART drugs, especially protease inhibitors, are substrates of the above transporters, leading to suboptimal drug concentrations inside microglia [24,25,26]. Therefore, it is important to develop a drug delivery strategy for ART drugs that crosses the BBB and bypasses efflux transporters in microglia, to improve viral suppression in the CNS. Previously, we reported a poly(lactic-*co*-glycolic acid) (PLGA)-based nanoformulation of elvitegravir (EVG, an FDA-approved integrase inhibitor for HIV-1) with ~95% loading efficiency, that efficiently suppressed HIV-1 in HIV-1-infected human-monocyte-derived macrophages, after crossing the in vitro BBB model [27]. These PLGA-based EVG nanoparticles (PLGA-EVG NPs) showed good biocompatibility with red blood cells and human primary macrophages. Compared with the EVG native drug, the PLGA-EVG NPs demonstrated ~50% higher BBB penetration in an in vitro BBB model. We also identified the mechanistic contribution of P-gp in interfering with the penetration of EVG in the in vitro BBB model and the capability of PLGA NPs to bypass BBB efflux transportation. Our objective in the current study was to evaluate the efficacy of our novel PLGA-EVG NPs in another, and perhaps more important, CNS HIV-1 reservoir, microglia. In the current study, we identified the cyto-compatibility and cellular internalization of the PLGA-EVG NPs in monocyte-derived microglia-like cells (MMG). We assessed the ability of PLGA-EVG NPs to suppress HIV-1 replication in MGM after crossing an in vitro BBB model. We also determined the ability of PLGA-EVG NPs to cross the BBB in immunodeficient *NSG* mice and performed a pilot study, to demonstrate HIV-1 suppression in the CNS, using an HIV-1 encephalitic (HIVE) mouse model.

## 2. Material and Methods

### 2.1. Materials

Poly(d,l-lactide-*co*-glycolide) (PLGA) (50:50 lactide-glycolide ratio, Mw: 31,000–50,000, ester-terminated) was obtained from Birmingham Polymers (Pelham, AL, USA). HPLC-grade acetonitrile (A955) and formic acid (85178), BD PrecisionGlide 25G needle (14-826-49), and BD 1 mL TB syringe (14-826-88) were obtained from Fisher Scientific (Hampton, NH, USA). EVG (E509000) was purchased from Toronto Research Chemicals, Inc. (Ontario, Canada). Sterile phosphate-buffered saline (PBS) (10100-031) was obtained from Gibco (Dublin, Ireland). Ethylenediaminetetraacetic acid (EDTA) (BM-150) was bought from Boston Bio Products (Ashland, MA, USA). Roswell Park Memorial Institute (RPMI) 1640 media (15-040-CV), lymphocyte separation medium (25-072-CV), l-glutamine (25-005-CI), and penicillin-streptomycin (P/S) (30-001-CI) were bought from Corning, Inc. (Tewksbury, MA, USA). Dulbecco’s Modified Eagle’s Medium (DMEM) (30-2002) was obtained from American Type Culture Collection. Fetal bovine serum (FBS) (S11150H) was obtained from Atlanta biologicals (Atlanta, GA, USA). The human recombinant cytokines, including M-CSF (300-25-100), GM-CSF (300-03), beta-nerve growth factor (NGF-β) (450-01), and CCL2 (300-04), used for microglia differentiation, were purchased from PeproTech (Rocky Hill, NJ, USA). Recombinant Human IL-2 Protein (202-IL) was purchased from R&D system, Inc. (Minneapolis, MN, USA). HIV-1 Ada strain was obtained from the National Institutes of Health (NIH) AIDS Reagent Program (Germantown, MD, USA). The P24 ELISA kit (801111) was purchased from ZeptoMetrix Corp (Buffalo, NY, USA), to assess HIV viral load in HIV-1-infected microglia. All other chemicals, including poloxamer 188 (pluronic F-68) (P1300, Mw: 8350), polyvinyl alcohol (PVA) (363138, Mw: 30,000–70,000), poly-L-lysine (PLL) (Mw: 30,000–70,000), coumarin-6 (442631), acetone (650501), nocodazole (M1404), cytochalasin D (C8273), chlorpromazine (C1240), monensin sodium salt (M5273), genistein (G6649), methyl-β-cyclodextrin (Mβ-CD) (C4555), hexadimethrine bromide (polybrene) (107689), MitoTrackerTM Deep Red (M22426), Texas RedTM Conjugate (T2875), and CellLightTM Late Endosomes-RFP (C10589), were obtained from Sigma-Aldrich Co. (St. Louis, MO, USA).

### 2.2. Preparation of PLGA-EVG NPs

PLGA-EVG NPs were generated as described previously, with 10% wt/wt loading of EVG to PLGA [28]. In brief, EVG and PLGA were dissolved completely in acetone and added dropwise into a 1% PVA aqueous solution. The mixed solution was placed on a magnetic stir plate, at 400 rpm, to form the NP suspension. The NP suspension was then added with PLL and poloxamer 188 and stirred overnight, to allow acetone evaporation. The non-uniform and larger aggregates of PLGA, PLGA-EVG, PVA, and PLL ingredients were removed by centrifugation, at 1000 rpm, for 10 min. The PLGA-EVG NP formulation was stored at either 4 °C for short-term use or at 20 °C for long-term use.

### 2.3. Generation of HIV-1-Infected Monocytes-Derived Microglia-Like Cells (MMG)

Human-monocyte-derived microglia-like cells (MMG) were differentiated from de-identified human blood, which was obtained from Interstate Blood Bank, Inc. (Memphis, TN), upon approval from the Institutional Review Board (IRB, UTHSC). Peripheral blood mononuclear cells (PBMCs) were isolated as described before [27]. Isolated PBMCs were cultured in RPMI media containing 1% l-glutamine and 5% penicillin–streptomycin. After overnight incubation, non-adherent cells were washed by PBS and cultured in a new flask. A human recombinant cytokine mixture containing M-CSF (10 ng/mL), GM-CSF (10 ng/mL), NGF-β (10 ng/mL), and CCL2 (100 ng/mL) was added to all the flasks, for microglia differentiation. Mature MMG were collected after 14 days of differentiation, when they reached 70–80% confluence. One T75 flask of MMG was plated in one 12-well plate, with an equal number of cells in each well. MMG were incubated with polybrene (2 µg/mL) and HIV-1 Ada strain at 3 ng/well for HIV-1 infection. After overnight incubation, MMG were washed twice with PBS, to remove the polybrene and HIV-1 Ada strain. After 7–10 days of the initial HIV-1 infection, cell culture media samples from MMG were collected, to assess HIV-1 p24 levels, using a p24 ELISA kit, to confirm the HIV-1 infection.

### 2.4. Biocompatibility Assay with MMG

A biocompatibility assay with MMG was performed in the presence of the EVG native drug (0–20 µM), or equivalent concentrations of PLGA-EVG NPs, and a human serum-bound NP (HS@PLGA-EVG), using an XTT cell viability kit (Cell signaling, Danvers, MA), as described before [27]. Briefly, uninfected MMG cells were incubated with tested compounds for 24 h, incubated with XTT detection solution, and absorbance was measured by using a plate reader, at 450 nm. The percentage of viable cells from treatment groups was calculated by comparing the absorbance with that of untreated cells. Data presented are from five replicates.

### 2.5. Cellular Uptake, Subcellular Localization, and Internalization Mechanism of PLGA NPs

The cellular uptake and internalization studies were performed by using uninfected MMG and seeded in either 6-well plates or cell culture chamber slides (CellTreat Scientific Product, Pepperell, MA), at a density of 8 × 10^5^ cells/well, as described previously [27]. To track or identify NPs in cells, NPs were tagged with a fluorescence dye, coumarin-6 (C6). The C6 labeling was achieved by the encapsulation process in PLGA NPs (PLGA-C6 NPs), in a similar manner as drug loading in PLGA-EVG NPs. To identify the endocytosis pathway, MMG were pretreated with various endocytosis inhibitors, including nocodazole (10 µg/mL), Cyto-D (10 µg/mL), CPZ (10 µg/mL), monensin (200 nM), genistein (200 µM), and MβCD (1 mM) for 30 min, and they were subsequently incubated with 2.5 µg/mL of PLGA-C6 NPs for 2 h. Semi-quantitative measurements of PLGA-C6 NP uptake in MMG cells were obtained by using the Accuri C6 Flow Cytometer (Accuri Cytometer, Inc., Ann Arbor, MI). To obtain the image of cellular internalization, the above experiment was also performed in a cell culture chamber slide obtained from CellTreat Scientific Product (Pepperell, MA), to be observed using a confocal microscope. After the experiment, cells were fixed, permeabilized, and mounted in Vectashield Mounting Medium (Vector Labs, Burlingame, CA), which contained DAPI (Life Technologies), to visualize nuclei. All the images were taken by using a laser confocal microscope (Carl Zeiss LSM 710, Oberkochen, Germany), under 400× magnification, using an oil immersion objective. PLGA-C6 NPs were also used to visualize the subcellular localization of the NPs by incubating together with markers of mitochondria (MitoTrackerTM Deep Red) at 30 nM, early endosome (Texas RedTM Conjugate) at 50 nM, late endosome (CellLightTM Late Endosomes-RFP) at 50 nM, and lysosome (LysoTrackerR Red DND-99, Life Technologies) at 75 nM for 2 h, as described previously [27]. The subcellular localization was observed by using confocal microscopy.

### 2.6. Viral Suppression of PLGA-EVG NPs in MMG after Crossing an In Vitro BBB Model

To determine the efficacy of PLGA-EVG NPs on viral suppression, we used HIV-1-infected primary MMG to create a modified in vitro BBB model in a Transwell^®^ plate, as described before [27]. Briefly, mouse astrocytes (C8-D1A and CRL-2541) and mouse endothelial cells (bEnd.3 and CRL-2299) were co-cultured in a Transwell^®^ -COL collagen-coated 0.4 μm pore polytetrafluoroethylene membrane plate (Sigma-Aldrich) at 2 × 10^4^ cells/well, to form an in vitro BBB model. After the BBB cell layers achieved ~90% confluency, the upper inserts containing confluent brain endothelial cells were transferred to a plate containing HIV-1 infected MMG. The upper inserts were exposed to control, blank PLGA NPs, EVG native drug (5 µg/mL), and PLGA-EVG NPs (5 µg/mL), for 7 days. HIV-1 viral loads from MMG cells were measured every day, using a p24 ELISA kit from the culture media of the bottom chamber. The upper inserts were replaced with fresh BBB cells after 3 days, to keep the BBB monolayer confluent. All the HIV-1 related experiments were performed in the BSL-3 laboratory in the Regional Biocontainment Laboratory (RBL) of UTHSC.

### 2.7. EVG Level in the In Vivo Mouse Model

Male *NOD.Cg-Prkdc^scid^Il2rg^tm1Wjl^/SzJ* (*NSG*) mice were purchased from Jackson Laboratory (Bar Harbor, MA) and were acclimated to the animal facility for at least 7 days. The average weight of studied mice was 30 g at the age of 10–12 weeks. A total of 16 mice were divided into two groups. One group of 8 mice was injected intraperitoneally (i.p.) with 20 mg/kg EVG native drug in 0.1–0.2 mL of PBS. Another group of 8 mice was injected i.p. with 20 mg/kg of PLGA-EVG NP in 0.1–0.2 mL of PBS. Mouse blood was collected at 3, 9, 24, 48, 72, and 96 h, in EDTA-containing blood-collection tubes, following the serial tail sampling procedure [29]. Mouse blood was settled down at room temperature and then centrifuged at 6000 rpm, for 10 min, for plasma harvesting. After blood collection, the mice were euthanized by exsanguination under deep isoflurane anesthesia, followed by cervical dislocation at 96 h. Then, the brain tissues were harvested. Mice plasma and brains were placed into tubes and frozen at −80 °C, until further analysis by LC–MS/MS. All experimental protocols involving the use of laboratory animals were approved by the UTHSC Institutional Animal Care and Use Committee (IACUC).

### 2.8. Quantification of EVG, Using LC–MS/MS

Mouse plasma and brain samples were analyzed for EVG concentration, using the same LC–MS/MS method as described before [30]. Briefly, EVG and internal standard (RTV) were quantified, using a tandem mass spectrometer AB SCIEX Triple Quad 5500 that was equipped with electron spray ionization. The isolation of analyzed compounds was conducted in a Shimadzu liquid chromatographic system (Kyoto, Japan). The sample extraction was performed by adding 9-volume of cold acetonitrile to an aliquot of plasma or brain homogenate. The multiple reactions monitoring (MRM) transitions (m/z) Q1/Q3 selected for quantitative analyses were 447.9/343.8 for EVG and 721.3/296.1 for the RTV.

### 2.9. Viral Suppression of PLGA-EVG NPs in HIV-1 Encephalitic (HIVE) Mice

Four-week-old male *NSG* mice were purchased from The Jackson Laboratory (Bar Harbor, ME). All experimental protocols involving the use of laboratory animals were approved by the IACUC of the University of Nebraska Medical Center and the NIH. To create the HIV-1-infected mouse model, 6 *NSG* mice were injected bilaterally into the basal ganglia with 5 × 10^5^ cell suspension in 5 μL containing HIV-1 ADA-infected MDM/MMG [31]. Fourteen days after the MDM/MMG injection, the animals were injected i.p. with vehicle control (PLGA-Blank NPs), EVG native drug, or PLGA-EVG NPs, in 0.1–0.2 mL of 5% dextrose, divided in three doses on the first day, to reach the dose of 200 mg/kg (*n* = 2 in each group). Mice were sacrificed on day 5 after the drug injection. Brain samples were collected and used for RNA extractions, and CNS HIV-1 p24 levels were assessed by measuring HIV-1 *gag* RNA levels, using reverse transcription polymerase chain reaction (RT-PCR). *GAPDH* was used as a housekeeping gene in this RT-PCR analysis.

### 2.10. Statistical Analysis

**Figure 1:**
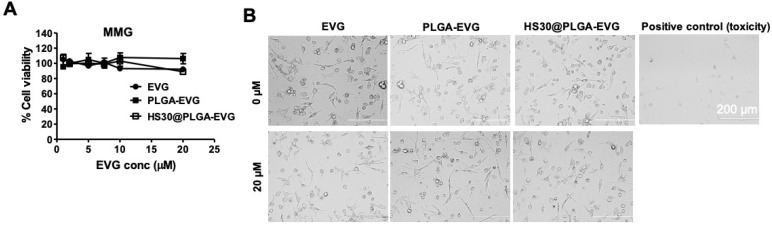
Biocompatibility of PLGA-EVG NPs in MMG. (**A**) The percentage of cell viability of MMG exposed with 0–20 µM test compounds include EVG, PLGA-EVG NPs, and 30% HS bound-PLGA-EVG NPs complexes (HS30@PLGA-EVG), measured by XTT assay. (**B**) Microscopic images of MMG with negative controls, positive control, and the highest concentration of EVG (20 µM) used in the cell viability assay. As a positive control, 5% acetonitrile was used. Mean ± SEM values are from five replicates.

**Figure 2:**
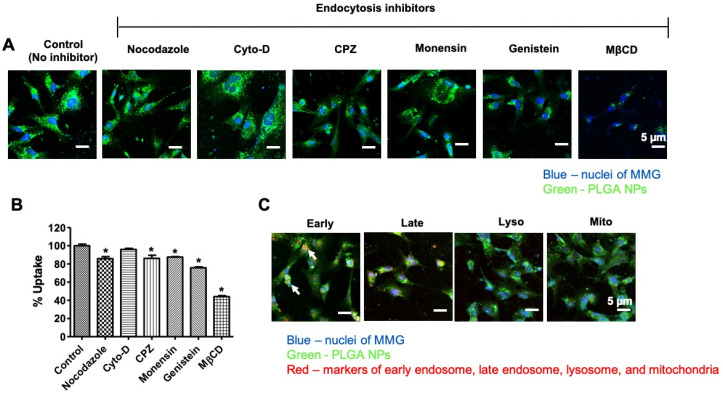
Internalization mechanism of the PLGA-C6 NPs in MMG. (**A**) Confocal images of cellular uptake of PLGA NPs (green) in MMG (blue nuclei), in the presence of various endocytosis inhibitors. (**B**) Relative cellular uptake percentage calculated from the mean fluorescence intensity measured by flow cytometry in MMG. Mean ± SEM are from three measurements. * *p* < 0.05. (**C**) Confocal images of sub-localization of PLGA-C6 NPs (green) in MMG (blue nuclei), in the presence of early endosome, late-endosome, lysosome, and mitochondria markers (red). Cells were visualized under 400× magnification (Bar = 5 µm).

**Figure 3:**
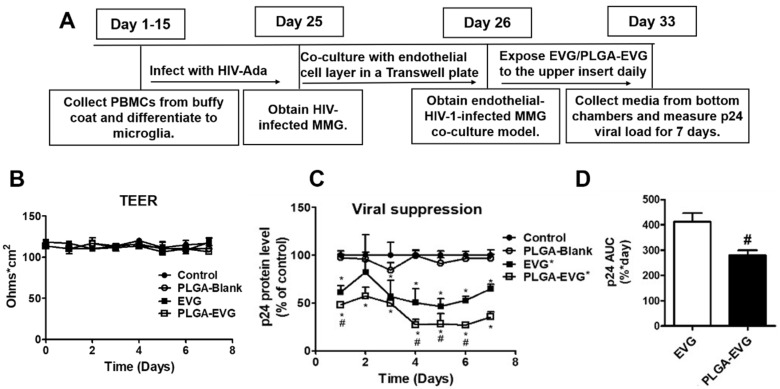
Viral suppression of EVG and PLGA-EVG in HIV-1-infected MMG after crossing an in vitro BBB model. (**A**) Experiment design to perform viral suppression study in MMG in an in vitro BBB model. (**B**) Daily BBB membrane integrity presented as TEER values. (**C**) Patterns of viral dynamics in HIV-1-infected MMG, with treatment response of EVG, PLGA-EVG, and PLGA-Blank NPs after crossing the in vitro BBB model. The p24 levels were normalized to the control MMG and reported as a percentage of the control group. (**D**) Treatment efficacy comparison of EVG and PLGA-EVG NPs presented as Area under the viral dynamic curve AUC_(0–t)_. Mean ± SEM values were graphed from triplicate samples, with MMG derived from one donor. * Indicates *p* < 0.05 compared to control; ^#^ indicates *p* < 0.05 compared to EVG native drug.

**Figure 4:**
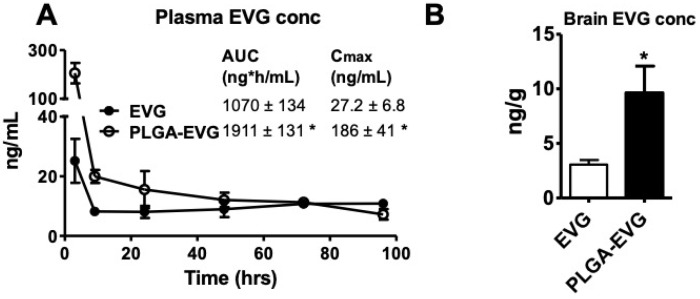
Plasma and brain concentrations of EVG and PLGA-EVG NPs in mice. (**A**) Plasma concentrations of EVG were measured after i.p. administration of a 20 mg/kg dose of native EVG (*n* = 8) or PLGA-EVG (*n* = 8) to mice by LC–MS/MS. Area under the curve (AUC) and maximum concentration (C_max_) were analyzed, using non-compartmental analysis by PK solver. (**B**) Brain concentrations of EVG were measured after sacrificing mice at 96-hour time point. Mean ± SEM values were graphed from eight mice measurements for each group. * Indicates *p* < 0.05 compared to EVG native drug.

All graphs and statistical analyses were performed by using GraphPad Prism 5 (GraphPad Software; La Jolla, CA). The statistical significance between two groups was determined by Student’s *t*-test analysis. The statistical significance among treatment groups with individual variable was determined by one-way ANOVA Tukey HSD post hoc test, which assumed that data was normally distributed with equal variance. The statistical significance among treatments groups with two variables was determined by using two-way ANOVA with a Bonferroni post hoc test, assuming normal distribution and equal variance. More specifically, Figure 3D and Figure 4D were analyzed with Student’s *t*-test. Figure 2B was analyzed with one-way ANOVA. Figure 1A, Figure 3B,C and Figure 4C were analyzed with two-way ANOVA. A *p*-value ≤ 0.05 was considered to be significant.

## 3. Results

### 3.1. Biocompatibility of PLGA-EVG NPs in MMG

In our previous report, we have shown a favorable safety profile of PLGA-EVG NPs with red blood cells and monocyte-derived macrophages [27]. Similarly, in order to safely use PLGA-EVG NPs in MMG, we evaluated the biocompatibility of PLGA-EVG NPs with MMG. Because human serum (HS) protein binding occurs when NPs are administered to human subjects [32], understanding the interaction of NPs with HS can predict their safety and efficacy profiles when used in the clinic. Additionally, we have shown the occurrence of human serum protein binding with our PLGA NPs [27]. Thus, we also performed the biocompatibility assay with protein-bound PLGA NPs. Neither PLGA NPs nor the protein-bound PLGA NPs changed the biocompatibility of EVG to MMG over the range of 0–20 µM. Approximately 100% cell viability was observed when incubated with EVG, PLGA-EVG, or 30% human serum bound EVG NPs (30HS@EVG NPs) (Figure 1A), with no observed cell morphology changes (Figure 1B).

### 3.2. Internalization Mechanism of the PLGA NPs in MMG

The endocytosis pathways of PLGA NPs in MMG were determined by a pretreatment with various endocytosis inhibitors. Microtubule-related internalization, macropinocytosis, clathrin-mediated endocytosis, lysosome-involved internalization, caveolae-mediated pathways, and caveolae-/clathrin-mediated endocytosis were assessed in the presence of endocytosis inhibitors, nocodazole, cytochalasin D (Cyto-D), chlorpromazine CPZ, monensin, genistein, and methyl beta-cyclo dextrin (M*β*-CD), which suppress encocytosis of PLGA-NPs, respectively [33,34]. Compared with the control, the cellular uptake of PLGA NPs was diminished by the compounds with the inhibition strength in the order of M*β*-CD > genistein > CPZ = nocodazole = monensin. Among these inhibitors, M*β*-CD inhibited up to 55% uptake of PLGA-C6 NPs, strongly suggesting that the main internalization of PLGA NPs in MMG occurred through caveolae-/clathrin-mediated endocytosis (Figure 2A,B). We further examined the subcellular fate of PLGA NPs, using a confocal microscopic analysis (Figure 2C). PLGA NPs were efficiently internalized in MMG after a 2 h exposure. A co-localization of early endosome markers (red) with PLGA NPs (green) was observed. No co-localization was observed in the presence of the lysosomal or mitochondria marker. This suggests that PLGA NPs can escape from lysosomal degradation and deliver therapeutics to the cells efficiently.

### 3.3. Improved Viral Suppression in HIV-1-Infected MMG after Crossing the In Vitro BBB

Previously, we have shown that PLGA-EVG NPs have a significantly lower viral load compared with the EVG native drug in HIV-1-infected macrophages after crossing an in vitro BBB [27]. Other than macrophages, microglia also serve as a sanctuary site for HIV-1 in the CNS, which cannot be suppressed efficiently [35]. A one-month treatment paradigm for collecting HIV-1-infected MMG and performing an EVG/PLGA-EVG viral suppression study was used, as described in the scheme (Figure 3A). Although the in vitro MMG is not representative of human primary microglia, MMG have shown to express microglia markers and possess a similar morphological structure as human primary microglia [36]. The endothelial monolayer was exposed to the drug on the top of HIV-1-infected MMG, and HIV-1 viral load was measured from the bottom chamber, which was not exposed to EVG directly. We did not observe monolayer integrity changes during the seven-day treatment, using transepithelial electrical resistance (TEER), a surrogate for transmembrane permeability (Figure 3B). PLGA-Blank NPs showed no effect on viral load, compared with control cells. Both EVG alone and PLGA-EVG NPs showed significant HIV-1 suppression in HIV-1-infected MMG after crossing the BBB on all the days, except day two for EVG alone, which was not significant compared to the control. Moreover, compared to EVG alone, PLGA-EVG showed significant HIV-1 suppression on day one, four, five, and six. Over the seven-day treatment, PLGA-EVG showed a greater extent of HIV-1 suppression, which suppressed ~50–70% of the virus, compared to EVG native drug, which suppressed ~20–50% of the virus. Moreover, PLGA-EVG NPs showed better efficacy for viral suppression on day one, four, five, and six, compared with the EVG native drug (Figure 3C, * *p* < 0.05). The p24 AUC showed the relative viral suppression for the EVG-free drug and PLGA-EVG NPs. PLGA-EVG NPs showed ~25% more viral suppression in HIV-1-infected MMG after crossing the in vitro BBB (Figure 3D, * *p* < 0.05).

### 3.4. EVG Levels in Mice

The plasma levels and brain permeability of EVG loaded on PLGA NPs were evaluated by comparing the plasma and brain concentrations of native EVG with PLGA-EVG NPs. We prepared calibration curves, using blank plasma and blank brain homogenates, to quantify plasma samples and brain samples, in order to minimize the matrix effect, respectively. The linear calibration curve was achieved over the range of 1–500 ng/mL, with a weighting factor of 1/y and a correlation coefficient (r2) of 0.999 for plasma EVG. The calibration curve for brain homogenates followed a linear regression over the range of 1–125 ng/mL, with a weight factor of 1/y and r2 of 0.997. The mean plasma concentration versus time profiles of PLGA-EVG NPs and native EVG after intraperitoneal (i.p.) injection is shown (Figure 4A, * *p* < 0.5). The calculated mean plasma C_max_ of PLGA-EVG NPs was 186 ng/mL, which is approximately six-fold greater than the C_max_ achieved by the native drug (27.2 ng/mL). The AUC(0-t) of the PLGA-EVG NPs was 1911 ng*h/mL, which is significantly higher than the native drug. The higher C_max_ and AUC_(0-t)_ indicate that the nanoformulation increases the bioavailability of EVG compared to the same dose of the free drug in mouse plasma. Mouse brain concentrations were also measured after sacrificing the mouse at 96 h, to analyze the permeability of EVG across the BBB. Brain EVG concentration was found to be ~two-fold higher for PLGA-EVG NPs compared to the native drug (Figure 4B, * *p* < 0.5). Overall, the data showed an improved delivery of EVG to the brain, using the PLGA NPs. Mice were healthy after injecting the EVG native drug/PLGA-EVG NPs. No adverse events, weight loss, or animal deaths were observed during the study. The enhanced efficiency of BBB transmigration observed with PLGA NPs is at least partially due to bypassing the efflux transporter, P-gp, as shown in our previous article [27].

### 3.5. Improved Viral Suppression in an HIV-1 Encephalitis (HIVE) Mouse Model

**Figure 5:**
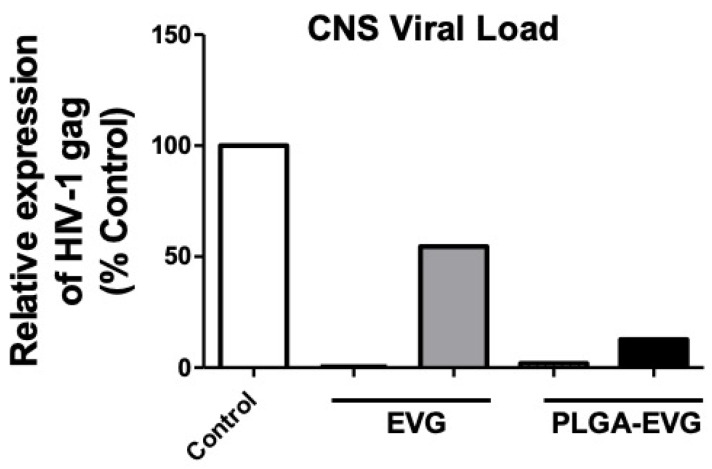
Viral suppression of EVG and PLGA-EVG in a HIVE mouse model. Viral suppression of EVG was measured after i.p. administration of a 200 mg/kg dose of vehicle control (PLGA-Blank NPs, *n* = 2), EVG native drug (*n* = 2), or PLGA-EVG (*n* = 2) to HIVE mice. CNS viral load was presented as *HIV-gag* level and normalized to the control group, which was injected i.p. with PLGA-Blank NPs, and reported as a percentage of the control group.

In our initial pilot experiment, we tested the PLGA-EVG NPs in an HIV-1 encephalitis (HIVE) mouse model where HIV-1-ADA-infected MDM were injected bilaterally into the basal ganglia of *NSG* humanized mice. In this experiment, each group of test compounds had *n* = 2 of mice. One mouse from the EVG group showed an almost undetectable CNS viral load, and another mouse showed ~50% decrease in viral load, compared with the control. In the PLGA-EVG group, one mouse showed an almost undetectable viral load and another mouse showed ~90% viral suppression. Although we could not do the statistical analysis due to the limited animal number, PLGA-EVG performed more consistently in both mice, with a trend of lower CNS viral load, compared with the EVG native drug (Figure 5). A further comprehensive investigation will be conducted in the future, to validate the efficacy of PLGA-EVG NPs in suppressing CNS viral load and improving cognitive dysfunction.

## 4. Discussion

This study demonstrated that a PLGA-based NP is an efficient delivery approach for EVG across the BBB. The PLGA-EVG NPs demonstrated a favorable safety profile with MMG. PLGA-EVG NPs showed an improvement in viral suppression in HIV-1-infected MMG after crossing the in vitro BBB, compared with the EVG native drug. The PLGA-EVG NPs demonstrated an improved plasma drug concentration and drug brain concentrations in an in vivo mouse model, relative to EVG alone. PLGA-EVG NPs also showed a trend of decreasing viral load in the CNS in the humanized HIVE mice model, as compared with the EVG native drug. To the best of our knowledge, this is the first report of using an EVG nanoformulation as a potential delivery method to cross the BBB and suppress HIV-1 in CNS microglia reservoirs.

Unlike macrophages and monocytes, which have been studied in depth with NP-based drug delivery for anti-HIV agents [37], the feasibility of delivering anti-HIV agents by using an NP-based drug delivery strategy to microglia in the brain has been studied in only a few reports. For example, Aalinkeel et al. investigated the feasibility of delivering galectin-1 by using gold NPs (Au-NP) to form a multivalent complex (Au-Gal-1) [38]. Au-Gal-1 significantly decreased the cell migration by up to 86%, indicating a five-fold increase in bioavailability. More importantly, the delivered galectin-1 successfully shifted microglia in the brain from polarized M1 to the unpolarized M2 form, which is a very critical step forward in treating HAND. Raymond et al. investigated the delivery of the negative factor (Nef) peptides, using magnetic NPs [39]. Nef peptides have the ability to reduce the release of Nef-containing exosomes in Nef-transfected microglia. Hence, BBB integrity will not be compromised due to Nef-containing exosomes. Exosomes belong to the family of extracellular nanovesicles and have a negative impact on BBB integrity in this specific case. Using magnetic NPs to deliver Nef peptides, they successfully improve the BBB integrity by 30% TEER value, indicating a target delivery of Nef peptides into the brain. Rodriguez et al., from the same group, investigated the delivery of siRNA targeting Beclin1, using a ferric-cobalt electromagnetic nanomaterial (CoFe2O4@ BaTiO3; MENP) [40]. The complex, MENP-SiBeclin1, was proved to cross the BBB, suppress viral replication, and relieve neurotoxicity, without compromising BBB integrity. While SiBeclin1 alone did not cause a significant decrease in viral titer, the MENP-SiBeclin1 complex reduced the viral titer by 53.6 ± 1.2% at 24 h and by 30.3 ± 1.8% at 48 h. With respect to inflammation, the MENP-SiBeclin1 complex also reduced the release of MCP-1, IL-8, TNF-α, RANTES, and IL-6 at 24 h, as well as MCP-1, IL-6, and IL-8 at 48 h. Although the above studies showed the benefit and feasibility of delivering anti-HIV agents to microglia in the brain, none of the studies delivered ART drugs to reduce viral replication in microglia and to treat the root cause of HAND. Since ART drugs are considered the most effective treatment strategy to control the progress of HIV-1, and microglia serve as viral reservoirs in the brain [1,8,9], a novel delivery strategy, such as the use of NPs, is needed to treat HIV-1 in the CNS and improve ART drug concentrations in microglia.

In our study, we selected EVG as the therapeutic molecule in our nanoformulation because EVG has a better safety profile than other classes of ARVs [41], which would make it more appropriate to use as a CNS therapy approach. EVG has limited BBB penetration, showed as low as a ~0.3% cerebrospinal fluid–plasma drug concentration ratio in HIV-infected individuals [42]. To improve the efficacy of EVG, we encapsulated the drug into a polymer-based NP formulation, to increase its transmigration across the BBB and increase drug uptake in microglia, after entry into the CNS. With regard to NPs, we selected a PLGA-based NP because it is a biodegradable and FDA-approved material for human clinical applications [43]. PLGA NPs have been shown to exhibit improved delivery of therapeutic molecules across the BBB, with safe and minimal/tolerable off-site toxicity to cells [44]. Compared to magnetic NPs, which have been extensively studied to deliver ART drugs to the brain, polymer-based NPs, such as the PLGA-based NPs we used, are easy to prepare, composed of FDA-approved nontoxic NPs, and do not require the application of an external magnetic field [37]. PLGA-based NPs have shown improved BBB penetration for delivering ART drugs [45], improved viral suppression, and reduced toxicity of ART drugs in brain macrophages [27,46]. However, to the best of our knowledge, this is the first report of a study using PLGA-based NPs to deliver ART drugs to microglia for HIV-1 suppression. More importantly, PLGA-based subcutaneous injections have been approved by the FDA for up to 533 mg per dose [47]. With our innovative formulation to target neuronal HIV and our on-campus sterile injectable facilities [48], we expect that PLGA-EVG NPs have the potential to move forward to clinical use, under the FDA’s breakthrough therapy or other facilitated regulatory pathways [49,50,51].

Additionally, from our knowledge, this is the first report studying the mechanistic internalization of NPs in human microglia cells. Understanding the mechanism of internalization of NPs by the targeted cells can increase our knowledge of how these particles are taken up and are transported within cells [52]. The literature reports have suggested that polymeric NPs are taken up by mouse primary microglia mainly by clathrin-mediated endocytosis [53,54,55]. Similarly, we observed that caveolae-/clathrin-mediated endocytosis serves as the major pathways for human MMG uptake of PLGA NPs. Additionally, our design of NPs allows the PLGA NPs to escape endo-lysosomal degradation/secretion, which also helps the therapeutic to work effectively inside the cells [56].

Nanotechnology is likely to provide a new approach for delivery of ART drugs in the treatment of HAND. Size, charge, and surface modification of PLGA NPs can all contribute to successful CNS delivery [37]. In general, PLGA NPs with a size smaller than 200 nm have a better chance to cross the BBB via transcytosis through clathrin-mediated endocytosis. Since the endothelial cell layer is negatively charged, positively charged NPs can enter the BBB through adsorptive transcytosis, which is easier than for neutral or negatively charged NPs. On the other hand, neutral and negatively charged NPs also have benefits because of their reduced protein adsorption, leading to a longer circulation period. NPs can also be modified with ligands to target transport- or receptor-mediated transcytosis, such as via albumin transporters and glucose transporter 1 (GLUT1) [37].

Recently, a new in vivo mouse model, which reproduced many important characteristics of HIV, has been developed by our group [57]. Humanized mice transplanted with CD34+ hematopoietic progenitor cells were infected by HIV-1ADA strain. This CD34+ mouse model represents human microglia-like cells in the mouse brain and can be used to study microglia viral reservoirs, including CNS HIV-1 viral load and the effects of therapeutic interventions. In order to move forward, the efficacy of our PLGA-EVG NPs will be determined by using this new mouse model [57]. We will also study neurotoxicity and the effects of therapeutic interventions on cognitive functions in the mouse brain [57]. Since nanotechnology is a novel delivery method, we will also study the safety profile for up to six months, with respect to long-term adverse reactions, systemic toxicities, neurotoxicities, and the accumulation of nanomaterials in body. Additionally, these HIV-1-infected humanized mouse models can also be used to study cognitive behaviors, using a conditioned fear test to study contextual memory and a differential learning (DL)/reversal learning (RL) procedure to study cognitive flexibility, to further study the cognitive behavior improvement. Furthermore, to move forward to clinical use, comprehensive toxicity and safety assessments of the nanoformulation need to be addressed, including animal studies using non-human primates to demonstrate efficacy, safety, and dose ranges.

**Figure 6:**
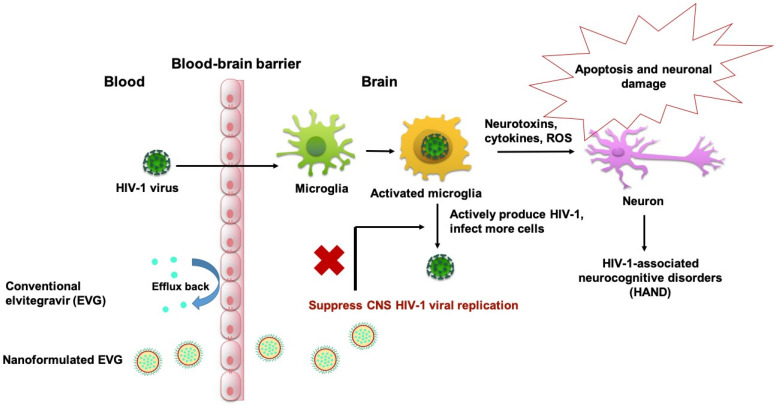
Graphic illustration representing the use of EVG nanoformulation across the BBB, to suppress the CNS HIV-1 viral replication in microglia cells.

Our current study demonstrated that a PLGA-based nanocarrier is an efficient delivery approach for EVG to cross the BBB and suppress HIV-1 viral replication in microglia. The PLGA-EVG NPs demonstrated a favorable biocompatibility profile with microglia cells. The PLGA-EVG NPs showed an improved viral suppression in HIV-1-infected microglia after crossing the in vitro BBB model. PLGA-EVG NPs also showed a higher accumulation in mouse brain tissue and a trend of decreasing CNS HIV-1 viral load in HIV-1-infected mice. As shown in Figure 6, the overall strategy for our study was to use nanoformulated EVG to bypass the efflux transporters on the BBB and deliver EVG successfully to the CNS. The EVG nanoformulation in the CNS suppresses active viral replication in microglia and subsequently reduces apoptosis and neuronal damage caused by activated microglia cells. The results we showed in this study will help us to create a safe and efficient drug delivery method, to target HIV reservoirs in the CNS, for potential clinical use.
